# Oroxylin A Accelerates Liver Regeneration in CCI_4_-Induced Acute Liver Injury Mice

**DOI:** 10.1371/journal.pone.0071612

**Published:** 2013-08-08

**Authors:** Runzhi Zhu, Guofang Zeng, Yinqin Chen, Qingyu Zhang, Bin Liu, Jie Liu, Hege Chen, Mingyi Li

**Affiliations:** 1 Laboratory of Regenerative Medicine, Department of Hepatobiliary Surgery, Affiliated Hospital of Guangdong Medical College, Zhanjiang, China; 2 Department of Pharmacology and Experimental Therapeutics, University of Maryland School of Medicine, Baltimore, Maryland, United States of America; Kaohsiung Chang Gung Memorial Hospital, Taiwan

## Abstract

**Introduction:**

Based on the previous research that oroxylin A can suppress inflammation, we investigated the hepatoprotective role of oroxylin A against CCl_4_-induced liver damage in mice and then studied the possible alteration of the activities of cytokine signaling participating in liver regeneration. Wild type (WT) mice were orally administrated with oroxylin A (60 mg/kg) for 4 days after CCl_4_ injection, the anti-inflammatory effects of oroxylin A were assessed directly by hepatic histology and indirectly by measuring serum levels of aspartate aminotransferase (AST), alanine aminotransferase (ALT) and Albumin. Proliferating cell nuclear antigen (PCNA) staining was performed to evaluate the role of oroxylin A in promoting hepatocyte proliferation. Serum IL-1β, TNF-α, IL-6 and IL-1Ra levels were measured by enzyme-linked immunosorbent assay (ELISA) and liver *HGF*, *EGF, TNF-α*, *IL-6*, *IL-1Ra* and *IL-1β* gene expression was determined by quantitative real-time PCR. The data indicated that the *IL-6* and *TNF-α* mRNA of oroxylin A administered group significantly increased higher than the control within 12 hours after CCl_4_ treatment. Meanwhile, oroxylin A significantly enhanced the expression of IL-1Ra at the early phase, which indicated that oroxylin A could facilitate the initiating events in liver regeneration by increasing IL-1Ra which acts as an Acute-Phase Protein (APP). In addition, a lethal CCl_4_-induced acute liver failure model offers a survival benefit in oroxylin A treated WT mice. However, oroxylin A could not significantly improve the percent survival of IL-1RI^−/−^ mice with a lethal CCl_4_-induced acute liver failure.

**Conclusions:**

Our study confirmed that oroxylin A could strongly promote liver structural remodeling and functional recovery through IL-1Ra/IL-1RI signaling pathway. All these results support the possibility of oroxylin A being a therapeutic candidate for acute liver injury.

## Introduction

The liver is a very important organ which regulates the balance of metabolic homeostasis, moreover, it has an amazing regenerative capability after liver injury [1], [2]. CCl_4_-induced acute hepatic injury widely used for studying liver regeneration [3], [4]. The hepatotoxicity of CCl_4_ specially causes oxidative stress and membrane damage [5], then lipid peroxidation induces hepatocellular damage and enhances inflammation. Hepatitis is fulminated within few hours after CCl_4_ treatment, which specifically leads to necrosis [6], [7].

**Figure 1:**
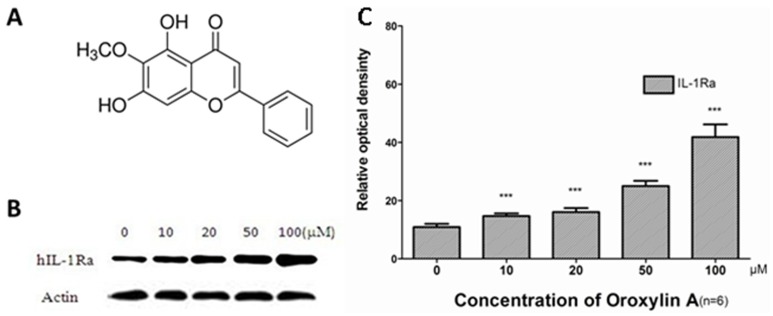
Oroxylin A up-regulates the production of IL-1Ra in hepG2 hepatoma cell line. (A) Molecular structure of oroxylin A (C_16_H_12_O_5_, MW: 284.). (B) Oroxylin A up-regulates the production of IL-1Ra in hepG2 hepatoma cell line (n = 6). (C) Inhibitory rate of IL-1Ra protein expression.

Oroxylin A (5, 7-Dihydroxy-6-methoxyflavone, C_16_H_12_O_5_, **Fig. 1A**) is a flavonoid isolated from *Scutellaria baicalensis*, which is one of the most important medicinal herbs in traditional Korean/Chinese/Japanese medicine. It has been used as a promising candidate for analgesic, anti-pyretic, anti-inflammation, anti-cancer, antiviral and anti-bacterial infections [8]. It has been reported that oroxylin A could suppress the growth of human cancer cells [9]–[11]. The hepatoprotective role of oroxylin A remains unclear. Previous studies indicated that oroxylin A could selectively induce apoptosis of cancer cells and inhibit normal hepatocyte apoptosis, which arouse our interesting to study whether oroxylin A could facilitate liver regeneration after injury.

The acute-phase proteins (APP) act as the hallmark of the acute-phase response, include a variety of secreted proteins the levels of which are modified during inflammatory conditions [12]. IL-1Ra is a very important APP in inflammation, which can modulate inflammatoty events and facilitate tissue repair. After liver injury, IL-1Ra is produced mainly by hepatocytes in response to several cytokines, such as IL-6 and TNF-α [13]. IL-1 plays a central role in inflammation, exerting its effects *in vitro* and *in vivo*, and often leading to sissue destruction. IL-1Ra is an anti-inflammatory protein, which competitively blocks the binding of IL-1α and IL-1β to type I IL-1 receptor. IL-1Ra has strong ability to inhibit the effects of IL-1α and IL-1β, and to reduce the severity of several inflammatory diseases [14]. HepG2 is a hepatoma cell line widely used to study the production of IL-1 family. In our previous study, the production of IL-1Ra could significantly increase after oroxylin A administration in hepG2 cells **(** **Fig. 1B and C** **, unpublished data)**. Previous evidence aroused our interest to study whether oroxylin A reduces inflammation through up-regulating the IL-1Ra expression in vivo. We hypothesized that oroxylin A possesses a potential antihepatotoxic activity to recover hepatocellular necrosis and accelerates liver regeneration during injury.

## Materials and Methods

### Animals and Chemicals

This study was carried out in strict accordance with the recommendations in the Guide for the Care and Use of Laboratory Animals of the Ministry of Health of the People’s Republic of China. The protocol was approved by the Committee on the Ethics of Animal Experiments of Guangdong Medical College. (Permit Number: SYXK (Guangdong) :2008-0007). All surgery was performed under sodium pentobarbital anesthesia, and all efforts were made to minimize suffering. We used only male C57 BL/6 mice (8 weeks old) to rule out gender differences, and mice were purchased from Shanghai Slac Laboratory Animal Corporation. Male IL-1RI−/− mice were kindly contributed by Dr.Yan Yu (Laboratory of Regeneromics, School of Pharmacy, Shanghai Jiaotong University, Shanghai.). The mice were maintained in a conventional clean facility in accordance with the National Animal Care and Use Committee. CCl_4_ and oroxylin A were purchased from Sigma-Aldrich Biotechnology (St Louis, MO, USA). Assays kits for the detection of serum alanine aminotransferase (ALT), aspartate aminotransferase (AST) and Albumin were purchased from Jiancheng Biological Technology, Inc. (Nanjing, China). Mouse monoclonal antibody against proliferating cell nuclear antigen (PCNA) and the SABC Staining Kit were from Boster Biological Technology (Wuhan, China). Serum IL-1β, TNF-α, IL-6 and IL-1Ra levels were measured by Enzyme-linked immunosorbent assay (ELISA) kit from R&D system (Minneapolis, MN, USA). All other chemicals were of the highest grade commercially available.

### Induction of Liver Injury and Oroxylin A Administration

Acute liver injury in mice was induced by injection of CCl_4_ at the dosage of 1 ml/kg body weight (1∶3 diluted in corn oil) intraperitoneally (i.p.). A lethal dose was administered by injection of CCl_4_ at the dosage of 2.6 ml/kg (1∶1 diluted in corn oil) i.p. At the indicated time points, serum and liver specimens were collected. Oroxylin A was dissolved in CMC-Na (Sodium Carboxymethylcellulose diluted in saline, 5 g/L) to 200 mg/ml final concentration for animal experiments and was dissolved in Dimethylsulfoxide (DMSO) to 100 mM final concentration for cell culture.

### Serum AST, ALT and Albumin

Serum AST, ALT and Albumin were determined with a commercial assay kit (Nanjing Jiancheng Biological Technology, Inc., China). Enzyme activities were shown in international unit per liter (IU/L).

### Histology-injury Grading

Formalin-fixed, paraffin-embedded liver sections were stained with hematoxylin-eosin for the histological investigations to evaluate the degree of necrosis after acute liver injury, which is based on severity of necrotic lesions in the liver parenchyma.

### Proliferating Cell Nuclear Antigen Staining

Proliferating cell nuclear antigen (PCNA) immunohistochemical staining was performed to evaluate hepatocyte proliferation. Liver tissues were fixed for 24 h in neutral buffered formalin, processed routinely and embedded in wax. Immunohistochemical staining was performed as previously described [15]. The sectioned liver tissues were stained using a mouse monoclonal antibody against PCNA and the SABC Staining Kit (Wuhan Boster Biological Technology, Wuhan, China) according to manufacturer’s protocol, then subjected to photomicroscopic observation (NIS-Elements Basic Research, Nikon Eclipse 50i, Kanagawa, Japan).

### Enzyme-linked Immunosorbent Assay

Serum IL-1β, TNF-α, IL-6 and IL-1Ra were measured by ELISA kit (R&D system, Minneapolis, MN, USA) according to the manufacturer’s instructions. ELISA was performed in triplicate for each sample.

### Real Time Quantitative PCR

Total liver RNA was prepared by using TRIZOL reagent (Invitrogen, Carlsbad, CA, USA). The quantification and qualification of RNA were performed using UV absorbance assay and electrophoresis in 1.2% agarose. RNA quality was satisfactory for the 28 s rRNA band on gel had twice the intensity of the 18 s rRNA band without significant smearing of rRNA. Real-time quantitative polymerase chain reactions were performed with the MJ chromo 4 RT-PCR detection system (Bio-Rad Laboratories, Hercules, CA, USA). Specific primers were designed using Primer 5.0 software (Premier Biosoft International, Palo Alto, CA, USA) and their sequences are listed in **Table 1**. As an internal control, the expression of the housekeeping gene *β-actin* was measured and remained constant during the experimental conditions in this study.

**Table 1:** Primer sequences used for real-time quantitative PCR.

Gene	Sense	Anti-sense
Mouse *IL-6*	CCACTCCCAACAGACCTGTCTATAC	CACAACTCTTTTCTCATTTCCACGA
Mouse *TNF-α*	AAGCCTGTAGCCCACGTCGT	CGTAGTCGGGGCAGCCTTGTC
Mouse *HGF*	GTGCTGGGCATTACTATGATGG	CTGCATCTCCCTCTCACACG
Mouse *EGF*	CGGACAGCTACACGGAATG	CGAGGCAGACACAAATAACCC
Mouse *IL-1β*	TGGTGTGTGACGTTCCCATT	CAGCACGAGGCTTTTTTGTTG
Mouse *IL-1Ra*	CTTTACCTTCATCCGCTCTGAGA	TCTAGTGTTGTGCAGAGGAACCA
Mouse *β-actin*	AGCCTTCCTTCTTGGGTATG	GTGTTGGCATAGAGGTCTTTAC

### Statistical Analysis

Student’s *t* test (unpaired, two-tailed) was used for comparisons between data from specified different conditions. Results from survival experiments were analyzed using the log-rank test and presented as Kaplan-Meier survival curves.

## Results

### Oroxylin A Protects Mice Against Acute Hepatocellular Damage

**Figure 2:**
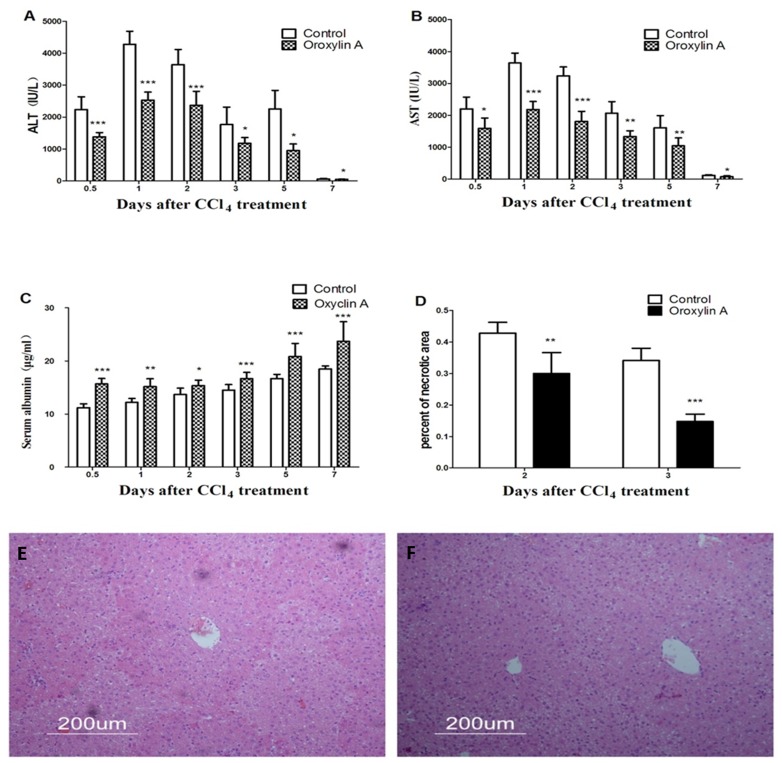
Oroxylin A protects liver against CCl_4_-induced acute liver injury. Mice were treated with CCl_4_ (1 ml/kg body weight and 1∶3 diluted in corn oil) to induce acute liver injury, then orally administered oroxylin A (60 mg/kg body weight and diluted in CMC-Na) 1 hour after CCl_4_ injection, once per day for 4 days. Control mice were treated with an equal volume of CMC-Na. Subsequently, serum ALT, AST and Albumin were measured at indicated time points and determined as described in materials and methods. (A) Serum alanine aminotransferase (ALT). (B) Serum aspartate aminotransferase (AST). (C) Serum Albumin. (D) Percentage of necrotic areas in ± oroxylin A groups were calculated at day 2 and day 3 after CCl_4_ treatment. (E) Representative liver section of control group was stained with hematoxylin and eosin (HE) at day 3 after CCl_4_ treatment, which shows partial necrosis with clusters of inflammatory cells around central vein. (F) Representative HE stained liver section of oroxylin A administration at day 3 after CCl_4_ treatment, which demonstrates inflammatory cells were decreased and histological recovery with only inconspicuous necrosis still remained around central vein; Values represent mean ± SE (n = 6). (*), (**), and (***) for *P*<0.05, *P*<0.01 and *P*<0.001 respectively.

To confirm the role of oroxylin A in protecting mice against hepatic damage, we used serum ALT, AST and Albumin as indicators for liver injury. After CCl_4_ treatment, serum ALT and AST rapidly elevated to peak level at day 1, then decreased thereafter, while oroxylin A treatment significantly inhibited the elevation of serum ALT and AST from day 1 to day 5 (**Fig. 2A and B**). The attenuated increasing of serum AST and ALT indicated that oroxylin A has a directly protective role on hepatocytes. Serum Albumin level is also considered as a very classical indicator for evaluating functional recovery of injured liver. In our study, we found that serum Albumin significantly increased after oroxylin A administration compared to the control (**Fig. 2C** **)**. To evaluate the effects of oroxylin A on hepatocellular necrosis and inflammation, histological changes in the liver after CCl_4_ treatment with or without oroxylin A administration were examined by hematoxylin-eosin staining. Liver sections from the oroxylin A administrated mice demonstrated only moderate necrosis involving the centrilobular areas, maintaining a rather normal architecture, the necrotic areas were significantly diminished around the central vein and centrilobular regions at day 3 after CCl_4_ treatment (**Fig. 2D, E and F**). These data together clealy indicated that Oroxylin A has potential anti-hepatotoxic activity.

### Oroxylin A Promotes Hepatocyte Proliferation from an Early Phase

**Figure 3:**
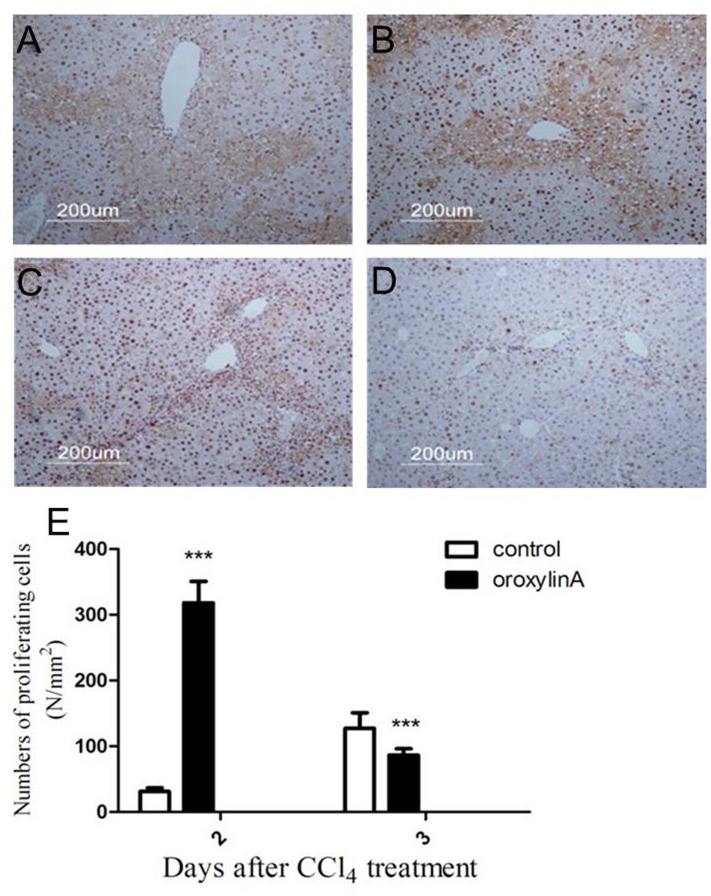
Oroxylin A promotes hepatic cell proliferation in CCl_4_-induced acute liver injury mice. Immunohistochemical staining for proliferating cell nuclear antigen (PCNA) was carried out as previously described. PCNA staining liver sections in the mice without (A) or with oroxylin A administration (B) at day 2 after CCl_4_ treatment. The control group showed few PCNA+ hepatocytes in centrilobular areas, but oroxylin A administration demonstrated numerous PCNA+ hepatocytes surrounding the edge of hepatocellular necrosis. PCNA staining liver sections from the control (C) and oroxylin A administration (D) at day 3 after CCl_4_ treatment. The control shows much more numbers of PCNA+ cells compared to oroxylin administration in the view. (E) PCNA+ cells in the liver sections were measured. At least six 12 mm^2^ tissue sections were counted for each mouse. Values represent mean ± SE (n = 6). (*), (**), and (***) for *P*<0.05, *P*<0.01 and *P*<0.001 respectively.

To confirm whetheroroxylin A has an advantage of accelerating hepatocyte proliferation from acute phase, we investigated the proliferation of hepatocytes by using immunostaining of PCNA in sections of liver tissue at day 2 and day 3 after CCl_4_ treatment. Compared to the control, oroxylin A administration dramatically increased the number of PCNA positive staining cells at day 2 after CCl_4_ treatment, a great number of PCNA^+^ hepatocytes could be detected surrounding the portal area (**Fig. 3A and B**). On the contrary, few PCNA^+^ hepatocytes were noticed in oroxylin A administration at day 3 after CCl_4_ treatment, which demonstrated that oroxylin A can significantly initiate liver regeneration at acute phase then rapidly terminate liver regeneration following functional recovery (**Fig. 3C and D**). PCNA^+^ cells in at least 12 mm^2^ tissue sections were counted for each mouse, and data shows that oroxylin A can accelerate hepatocyte proliferation (**Fig. 3E**).

### Serum Levels of IL-1β, TNF-α, IL-6 and IL-1Ra

**Figure 4:**
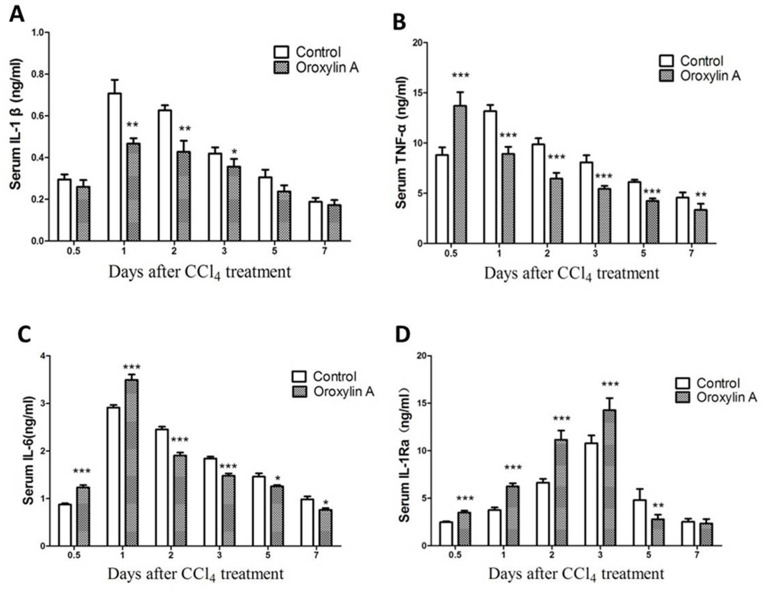
Serum levels of IL-1β, TNF-α, IL-6 and IL-1Ra (± Oroxylin A) after CCl_4_ treatment (1 ml/kg body weight). Serum were collected at indicated time points after CCl_4_ treatment, and serum levels of IL-1β, TNF-α, IL-6 and IL-1Ra were determined by using enzyme-linked immunosorbent assay (ELISA) kits according to manufacturer’s instructions. (A) Serum IL-1β. (B) Serum TNF-α. (C) Serum IL-6. (D) Serum IL-1Ra. Data indicates that oroxylin A significantly enhances the production of IL-1Ra in CCl_4_-induced acute liver injury mice. Values represent mean ± SE (n = 6). (*), (**), and (***) for *P*<0.05, *P*<0.01 and *P*<0.001 respectively.

To evaluate the hepatoprotective mechanism of oroxylin A, serum IL-1β, TNF-α, IL-6 and IL-1Ra levels were determined by ELISA kit. Serum IL-1β was found to be rapidly elevated after CCl_4_ treatment, which was supprted by previous reports [13], whereas oroxylin A administration resulted in significant attenuation of the elevation at day 1 after CCl_4_ treatment (**Fig. 4A**). CCl_4_-induced acute liver injury could activate hepatic non-parenchymal cells (including Kupffer cells and Stellate cells) and increase the production of TNF-α and IL-6 [16]. In our study, we found that serum TNF-α and IL-6 were rapidly increased and reached the peak level individually within 12 h/24 h in oroxylin A administration group compared to the control, and then decreased rapidly within 24 h/48 h (**Fig. 4B and C**). Oroxylin A administration up-regulates serum IL-1Ra gradually from an very early time and make it reached a peak level at day 3 after CCl_4_ treatment compared to the control (**Fig. 4D**).

### Gene Expression of *HGF, EGF, TNF-α, IL-6, IL-1Ra and IL-1β* in Liver

**Figure 5:**
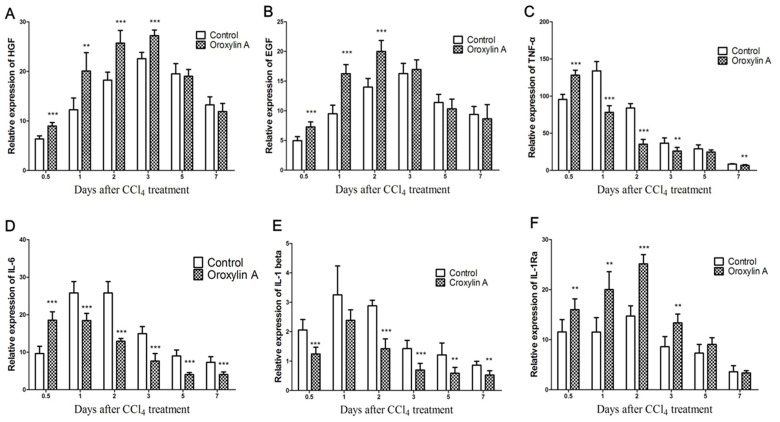
mRNA expression of *HGF, EGF, TNF-α, IL-6, IL-1β* and *IL-1Ra* (± Oroxylin A) after CCl_4_ treatment (1 ml/kg body weight). Total RNA in liver tissue of mice was isolated using TRIZOL reagent and quantified as described in materials and methods. (A) mRNA expression of *HGF*. (B) mRNA expression of *EGF*. (C) mRNA expression of *TNF-α*. (D) mRNA expression of *IL-6*. (E) mRNA expression of *IL-1β*. (F) mRNA expression of *IL-1Ra*. Data also shows that oroxylin A up-regulates the mRNA level of IL-1Ra in a CCl_4_-induced acute liver injury model. Values represent mean ± SE (n = 6). (*), (**), and (***) for *P*<0.05, *P*<0.01 and *P*<0.001 respectively.

Real time quantitative PCR was used to quantify the levels of *HGF, EGF, TNF-a, IL-6, IL-1Ra* and *IL-1β* mRNA in liver. Data showed that the production of *HGF, EGF TNFF-α*, *IL-6* and *IL-1Ra* mRNA was upregulated more rapidly in the oroxylin A administration group during the early phase and kept at a generally higher level within the process of liver regeneration (**Fig. 5A, B, C, D and E**).

### Oroxylin A Reduces Mortality after a Lethal Dose Performance through IL-1Ra/IL-1RI Phathway

**Figure 6:**
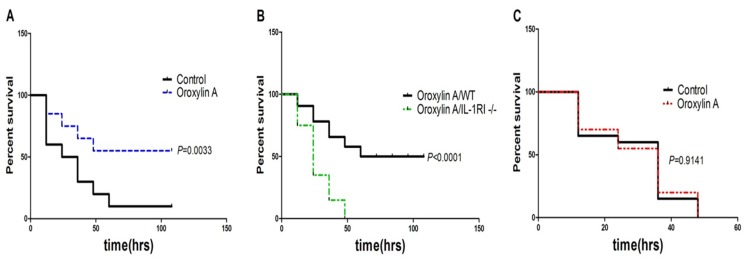
Oroxylin A increased probability of survival after a lethal dose of CCl_4_ treatment (2.6 ml/kg body weight). (A) WT mice were respectively administered with or without oroxylin A once per day for 5 days after CCl_4_ (2.6 ml/kg body weight) treatment (*P* = 0.0033). (B) After a lethal dose of CCl_4_ (2.6 ml/kg body weight) treatment, the probalility of survival in IL-1RI−/− mice significantly decreased compared to the WT mice (*P*<0.0001). (C) Oroxylin A administration could not improve survival of IL-1RI−/− mice after a lethal dose of CCl_4_ (2.6 ml/kg body weight) performace, which indicated that oraoxylin A protects against CCl_4_-induced acute liver failure directly through regulating IL-1Ra/IL-1RI pathway (*P* = 0.9141, non-significant). Survivals were scored twice per day, and the results were analyzed using the log-rank test and expressed as the Kaplan-Meier survival curves (n = 20).

In a previous experiment to see the dosage-dependent effect of CCl_4_, data indicated that 2.6 ml/kg CCl_4_ is a median lethal dose (mortality 50%, data not shown) within 24 hours in wild type mice. Oroxylin A oral administration offers a strong survival benefit for acute liver failure mice, increasing the probability of survival significantly from the early phase after CCl_4_ injection (**Fig. 6A**). More importantly, oroxylin A could not improve survival of IL-1RI−/− mice after a lethal dose performace (**Fig. 6B and C**), which indicated that oroxylin A could protect against CCl_4_-induced acute liver failure by regulating IL-1Ra/IL-1I pathway.

## Discussion

CCl_4_-induced liver injury has been used for decades because the elementary lesions caused by this hepatotoxin replicate those seen in most cases of human liver disease, which makes it be a good model to study signal transduction and cell cycle events *in vivo* [17], [18]. In previous studies, the pathological roles of CCl_4_ to the animal body is mainly restricted to liver, while lethality of high-does CCl_4_ is mostly related with acute liver failure instead of direct injury to other organs. In this study, we confirmed the protective role of oroxylin A against the typical acute liver injury. Oral administration of oroxylin A could significantly reduced mortality rate to wild type mice which received a LD_50_ dosage of CCl_4_. It is reasonable to hypothesize that administration of oroxylin A could reduce animal mortality mainly through attenuating acute liver damage by CCl_4_, and facilitating the preservation and restoration of liver functions. In our study, oroxylin A could up-regulate the production of IL-1Ra *in vitro* and *in vivo*. But it could not improve survival of IL-1RI−/− mice after a lethal dose performace, which indicated that oraoxylin A protects against CCl_4_-induced acute liver failure by regulating IL-1Ra/IL-1RI pathway. It is reported that IL-1RI-deficient mice exhibited an attenuate inflammatory response compared with wild type mice [19]. Serum aminotransferase activities have been utilized as the indicators of liver damage, including ALT and AST. These enzymes are released from damaged hepatocytes into the blood, which have been widely recognized as very important indicators to judge the severity of acute hepatic injury [20]. Data indicated that administration of oroxylin A attenuates the elevation of serum ALT and AST within 12 hours induced by CCl_4_ in mice. This time point is defined as the early stage of liver damage in which cell apoptosis and necrosis dominate the process. Furthermore, oroxylin A administration significantly improved serum level of Albumin, which also indicated that oroxylin could promote the functional recovery of liver [21]. Meanwhile, we used histological methods as supportive means to reveal the degree of cell necrosis and inflammation. Data also showed that oroxylin A oral administration inhibited inflammation, necrosis, and destruction of liver architecture.

Regeneration of liver gradually performs following liver damage, which was shown as cell proliferation rate would naturally increase till the original weight and shape of the liver is restored and liver functions recovery [1], [22], [23]. The density of positive cells in tissue section immunostained with PCNA antibody was used as a statistical index to measure the possible role of oroxylin A on the regeneration of liver. In our study, data strongly indicated that oroxylin A administration contributes to faster recovery after CCl_4_-induced liver injury by promoting the endogenous regeneration during the whole liver damage process.

To investigate the underlying mechanism, we evaluated the effects of oroxylin A administration on the serum levels of certain key cytokines tightly related with inflammation and cell proliferation. IL-1β, TNF-α, IL-6 and IL-1Ra, as acute-phase proteins, are considered to be biomarkers that reflect inflammatory conditions [24]. IL-1β plays a key role in inflammatory conditions, usually leading to tissue destruction. Furthermore, IL-1β has been previously shown to antagonize hepatocyte proliferation [25], [26]. Similar to IL-1β-deficient mice, IL-1RI-deficient mice had a reduced acute phase response to inflammatory conditions [27]. Serum IL-1β increases dramatically during inflammatory and non-inflammatory processes [14]. In the present study, we observed that oroxylin A administrated mice demonstrated lower serum level of IL-1β at day 1 after CCl_4_ treatment compared to the control. IL-6 and TNF-α expression has been identified as attractive targets for liver regeneration, TNF-α acts as a pro-inflammatory mediator in liver apoptosis, is also linked to cytotoxicity induced by CCl_4_ [28], [29]. Kupffer cells (macrophages in liver) produce TNF-α in rapid response to tissue injury, which up-regulates the expression of IL-6. TNF-α and IL-6 together activate neighboring hepatocytes, which causes signal transducer and activator of transcription STAT3 activation and the production of several other proteins that are shared within the growth-factor-mediated pathways. The mechanism of IL-6 and TNF-α in protecting the liver against injury has not been defined yet [30]–[32]. Previous studies showed that liver regeneration and hepatoprotection require the cytokine IL-6 after liver injury [33], [34], but overexpression of IL-6 causes liver injury [35], [36]. In our study, we found that TNF-α and IL-6 expression of oroxylin A administration rapidly reached a peak at Day 0.5 was then kept a relatively lower level at Day 1, 2, 3 and 5 compared to the control, which was considered that the levels of TNF-α and IL-6 perform gradually decreasing in later phase may facilitate liver regeneration. Approaching further into the mechanisms, we found that the *IL-6* and *TNF-α* mRNA of oroxylin A administration was enhanced in the similar pattern as the levels of corresponding proteins, leading to the conclusion that oroxylin A could indeed alter the expression of certain cytokines through IL-1Ra/IL-1RI pathway to regulate liver regeneration.

In addition, we measured the expression of growth factors such as HGF and EGF. It is confirmed that they could promote hepatic survival by stimulating liver regeneration and providing hepatoprotection in various models of liver injury [37]. And it has been proven that HGF and EGF are the main growth factors secreted after hepatic injury [23]. HGF is the most potent mitogen for mature hepatocytes and acts as a hepatotropic factor. Liver HGF expression increased markedly after various liver injuries such as hepatitis, ischemia, physical crush and partial hepatectomy. HGF also acts as a trigger for liver regeneration and strongly enhances EGF expression. Previous studies confirmed that the liver regenerative response is blocked if antibodies to HGF are administered after CCl_4_ treatment [38], [39]. Our data indicated that HGF and EGF expressions of oroxylin A administration significant increased higher during the early phase after CCl_4_ treatment compared to the control. Then HGF and EGF expressions reached even lower levels at Day 5 compared to the control, which indicated that the liver regeneration was terminated at a very earlier phase after oroxylin A administration.

**Figure 7:**
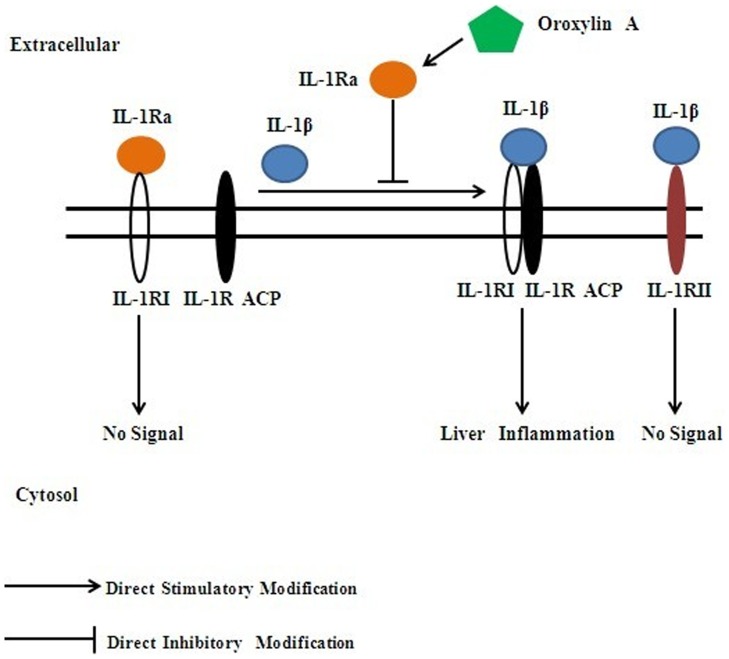
The underlying mechanism of oroxylin A accelerating liver regeneration. Oroxylin A up-regulates the production of IL-1Ra. Meanwhile, it acts as a negative regulator to IL-1β signaling by binding and blocking the fuctional receptor (IL-1 receptor type-I) without activation.

In conclusion, we found oroxylin A possesses strong beneficial effects against acute liver injury **(** **Fig. 7** **)**. Our results showed that oroxylin A could increase the production of IL-1Ra, IL-1Ra plays an anti-inflammatory role in acute liver inflammation, which could induce the expressions of inflammatory cytokines (IL-6 and TNF-α) markedly increasing at the very early stage, and then activate crucial signal transducers related to liver regeneration. Thereafter, the expressions of IL-6 and TNF-α were significantly alleviated to reduce inflammation. The following dramatic elevation of HGF and EGF could promote hepatic survival by stimulating hepatocyte regeneration. It is illuminated that the IL-1Ra/IL-1RI pathway plays an important role in the protective effects of oroxylin A against liver damage. The protective effect of oroxylin A inspires people of its clinical potential in the development of novel therapeutic candidate for acute liver injury.
